# Phosphonate-Functionalized Polycarbonates Synthesis through Ring-Opening Polymerization and Alternative Approaches

**DOI:** 10.3390/polym15040955

**Published:** 2023-02-15

**Authors:** Hien The Ho, Nam Hoai Nguyen, Marion Rollet, Trang N. T. Phan, Didier Gigmes

**Affiliations:** Aix Marseille Univ, CNRS, Chemistry Department, Institut de Chimie Radicalaire UMR 7273, Saint Jérôme Campus, 13397 Marseille, France

**Keywords:** ring-opening polymerization (ROP), phosphonate-functionalized polycarbonate, post-modification, “click” chemistry

## Abstract

Well-defined phosphonate-functionalized polycarbonate with low dispersity (*Ð* = 1.22) was synthesized using organocatalyzed ring-opening polymerization (ROP) of novel phosphonate-based cyclic monomers. Copolymerization was also performed to access different structures of phosphonate-containing polycarbonates (PC). Furthermore, phosphonate-functionalized PC was successfully synthesized using a combination of ROP and post-modification reaction.

## 1. Introduction

In the last decades, phosphorous polymers have been widely used in a variety of applications, including dental, energy, and oilfield applications [1,2,3,4]. Owing to their thermal stability properties, phosphonate-based polymers have become some of the most desired flame-retardant materials [5,6,7]. Furthermore, due to the high affinity of phosphonic acid with metallic ions, phosphonate-based polymers have also been transformed into phosphonic acid forms for water purification [8]. To date, the majority of these polymers have been obtained by incorporating the phosphonate group into macromolecular chains via the conventional or controlled radical (co)polymerization of various phosphonate-containing vinyl monomers [9,10,11]. As a result, these materials consist of vinylic polymer backbones that are difficult to degrade. This may be a significant concern for the environment or (bio)applications. To address this issue, there are a few examples of the synthesis of phosphonate-containing biodegradable polymers. For example, Ho and coworkers have reported the simple synthesis of well-defined phosphonate-terminated poly(ε-caprolactone) (PCL) using the organocatalyzed ring-opening polymerization (ROP) technique and phosphonate-containing hydroxyl molecule as the initiator [12]. Using a similar strategy, other phosphonate-terminated biodegradable polymers such as polylactide (PLA) and polycarbonate (PC) were also successfully synthesized [13,14]. In these studies, the phosphonic acid-terminated polymers were produced by dealkylation of the phosphonate group and then grafted onto the surface of oxide metal nanoparticles for various applications. While the synthesis of phosphonate-terminated biodegradable polymers has been thoroughly described, the introduction of pendant phosphonate groups into biodegradable polymers has rarely been reported. Thus, the synthesis of phosphonate-functionalized biodegradable polymers remains a significant challenge.

Owing to their biodegradability and biocompatibility, polycarbonates are considered as one of the most important biomaterials [15]. Recently, functional polycarbonates bearing different chemical functionalities have been utilized in various applications, including drug delivery, antimicrobial applications, and tissue engineering applications [15,16,17]. Although many functional PC have been synthesized, the synthesis of phosphonate-functionalized PC is still unknown. To our knowledge, only one attempt has been made to graft the phosphonic acid group on the PC chains using the coupling reaction between an amino-phosphonic acid molecule and carboxylic acid-based PC [18]. However, only partial functionalization was achieved, resulting in an undefined polycarbonate containing both pendants carboxylic and phosphonic acids. In this context, we developed different strategies for synthesizing phosphonate-functionalized polycarbonates. As a direct method, a novel phosphonate-based cyclic carbonate was synthesized and then served as a monomer for the ring-opening polymerization (ROP) to produce the corresponding well-defined phosphonate-functionalized PC. Furthermore, the synthesis of phosphonate-grafted PC was demonstrated using a combination of the ROP and “click” chemistry to compare the efficiency and versatility of the two methods.

## 2. Experimental Section and Methods

### 2.1. Materials

All reagents used in this study were purchased from Sigma-Aldrich (Saint Quentin Fallavier, France) unless otherwise noted. 2,2-bis(hydroxymethyl)propionic acid (bis-MPA, 98%), *p*-toluenesulfonic acid monohydrate (PTSA.H_2_O, >98.5%), 4-(dimethylamino)pyridine (DMAP, 99%), Dowex^®^50WX8 hydrogen form (50–100 mesh), ethyl chloroformate (97%), 8-diazabicyclo[5.4.0]undec-7-ene (DBU, 98%), benzoic acid (99.5%), acetone (99.9%), dichloromethane (DCM, 99.5%), tetrahydrofuran (THF, 99.9%), diethyl ether (99%), methanol (CH_3_OH, 99.8%), water (HPLC grade), *N*,*N*-dimethyl formamide (DMF, 99.9%), diethyl (3-bromopropyl)phosphonate (95%), copper(I) bromide (CuBr, 98%), *N*,*N*,*N*′,*N*″,*N*″-pentamethyldiethylenetriamine (PMDETA, 99%), sodium azide (NaN_3_, 99%), and dimethyl (2-hydroxyethyl)phosphonate (95%) were purchased from Acros Organics (Geel, Belgium). 2,2-dimethoxypropane (>98%), *N*,*N*’-dicyclohexyldicarbodiimide (DCC, >98%), and triethylamine (TEA, >99%) were purchased from TCI (Zwijndrecht, Belgium). Benzyl alcohol (BnOH, 99%, Alfa Aesar, Karlsruhe, Germany) and dialysis membrane (Standard RC, 3500 Da, Spectrum Laboratories, Racho Dominguez, CA, USA) were used as received. 1-(3,5-bis(trifluoromethyl)phenyl)-3-cyclohexylthiourea (TU) and 5-methyl-5-propargylxycarbonyl-1,3-dioxane-2-one (MPC) were synthesized according to the literature procedures [19,20,21].

### 2.2. Characterization

#### 2.2.1. High-Resolution Mass Spectrometry (HRMS)

Experiments were performed using a Synapt G2 HDMS quadrupole/time-of-flight (Manchester, UK). Samples were introduced at a 10 µL min^−1^ flow rate (capillary voltage +2.8 kV; sampling cone voltage varied between +20 V and +60 V) under a desolvation gas (N_2_) flow of 100 L h^−1^ heated at 35°C. Accurate mass experiments were performed using reference ions from PPG, PEG, or CH_3_COONa internal standard. All the samples were dissolved in methanol doped with 3 mM ammonium acetate before analysis. Data analyses were conducted using MassLynx 4.1 programs provided by Waters.

#### 2.2.2. Fourier-Transformation Infrared (FT-IR)

Spectra were recorded using a Perkin Elmer Spectrum Two FT-IR Spectrometer with an ATR accessory.

#### 2.2.3. Nuclear Magnetic Resonance (NMR)

^1^H, ^13^C, and ^31^P NMR spectra were recorded on a Bruker AC 400 MHz using deuterated solvents such as CDCl_3_ or acetone D6. Chemical shifts were reported in parts per million (ppm) calibrated using residual non-deuterated solvent as internal reference (CDCl_3_ at δ 7.26 ppm (^1^H NMR) and δ 77.16 ppm (^13^C NMR); acetone D6 at δ 2.05 ppm (^1^H NMR) and δ 29.84 ppm (^13^C NMR)).

#### 2.2.4. Size Exclusion Chromatography (SEC)

Polymers were characterized using the PL120 system (Polymer Laboratories, Church Stretton, UK), equipped with an injection valve (50 μL loop volume), a column oven, and a refractive index detector thermostated at 50 °C. The stationary phase was a set of two linear M PSS GRAM (300 × 8 mm) columns (PSS, Mainz, Germany). The eluent was DMF supplemented with NaNO_3_ (50 mM) and delivered at a 1 mL/min flow rate. Samples were solubilized in a mixture of the eluent and toluene (flow marker) at 0.25 vol.% and at a concentration of 0.25 wt%. Polymer equivalent number-average molar masses (*M*_n,SEC_) and dispersity (*Ɖ*) were calculated using a poly(methyl methacrylate) (PMMA) calibration curve based on PMMA standards from 0.885 to 520.0 Kg·mol^−1^ (Agilent, Santa Clara, CA, USA).

### 2.3. Synthesis of 2,2,5-Trimethyl-1,3-dioxane-5-carboxylic Acid (Acetonide-bisMPA, **1**)

In a 250 mL round bottom flask equipped with a magnetic stirrer, 2,2-bis(hydroxymethyl)propionic acid (bis-MPA, 32.00 g, 0.239 mol), acetone (120.0 mL), and 2,2-dimethoxypropane (30.0 mL, 0.244 mol) were sequentially added and stirred under argon at room temperature. Then, the solution of *p*-toluenesulfonic acid monohydrate (PTSA.H_2_O, 0.40 g, 2.11 × 10^−3^ mol) in acetone (5.0 mL) was injected into the reaction flask. After 90 min, 2,2-dimethoxypropane (20.0 mL, 0.163 mol) was added and stirred to obtain a homogenous solution. The reaction was continuously stirred at room temperature for 180 min. To stop the reaction, the solution of saturated sodium bicarbonate (NaHCO_3_, 30.0 mL) was added and stirred for 2 min. The mixture was concentrated before being extracted with diethyl ether (2 × 80.0 mL). The organic phase was then washed with brine solution (2 × 40.0 mL), dried with sodium sulfate (Na_2_SO_4_), filtered, and concentrated under reduced pressure at 40 °C to obtain the white solid (33.82 g). Yield: 81.3%.

^1^H NMR (400 MHz, acetone D_6_) δ (ppm): 4.15 (-OC**H**_2_-, d, *J* = 11.7 Hz), 3.65 (-OC**H**_2_-, d, *J* = 11.7 Hz), 1.39 and 1.33 (-C**H**_3_ of acetonide), and 1.19 (-C**H**_3_, s). ^13^C NMR (101 MHz, acetone D_6_) δ (ppm): 174.80 (-**C**OOH), 97.50 (-O**C**(CH_3_)_2_O-), 65.58 (-O**C**H_2_-), 41.07 (**C**(CH_3_)COOH), 23.76 and 22.52 (-**C**H_3_ of acetonide), and 18.08 (-**C**H_3_).

### 2.4. Synthesis of 2-(Dimethoxyphosphoryl)ethyl 2,2,5-trimethyl-1,3-dioxane-5-carboxylate (**2**)

In a 250 mL round bottom flask equipped with a magnetic stirrer, a solution of *N,N’*-dicyclohexyldicarbodiimide (DCC, 22.70 g, 0.11 mol) in DCM (50.0 mL) was added dropwise into a solution of acetonide-bisMPA **1** (18.0 g, 0.103 mol), dimethyl (2-hydroxyethyl)phosphonate (15.52 g, 0.101 mol), and 4-(*N*,*N*-dimethylamino)pyridine (DMAP, 0.72 g, 5.90 × 10^−3^ mol) in DCM (90.0 mL) at 0 °C under argon. After this, the solution was stirred at 0 °C for 3 h and then stirred at room temperature for 2 days. The mixture was filtered to remove the white solid. The filtrate was then washed with HCl (1 M, 60.0 mL), saturated NaHCO_3_ (60.0 mL), deionized water (60.0 mL), and brine solution (2 × 80.0 mL). The organic layer was dried over Na_2_SO_4_, filtrated, and concentrated under reduced pressure. The crude oil was dissolved in diethyl ether (25.0 mL) and cooled at −20 °C for 3 h. The solid precipitate was removed by filtration. The solvent was then removed using a rotary evaporator at 45 °C to give a viscous transparent oil. The crude product was purified using silica gel column chromatography with eluting solvent ethyl acetate (100%) and methanol/ethyl acetate (1/10: *v*/*v*) to give a colorless and viscous oil (21.62 g). Yield: 69%.

^1^H NMR (400 MHz, acetone D_6_) δ (ppm): 4.32 (-COOC**H**_2_-, dt, *J* = 14.3, 7.2 Hz), 4.17 (-OC**H**_2_-, d, *J* = 11.7 Hz), 3.73 (-PO(OC**H**_3_)_2_, d, *J* = 10.9 Hz), 3.64 (-OC**H**_2_-, d, *J* = 11.7 Hz), 2.20 (-C**H**_2_PO(OCH_3_)_2_, dt, *J* = 18.5, 7.2 Hz), 1.43 and 1.26 (-C**H**_3_ of acetonide), and 1.19 (-C**H**_3_, s). ^13^C NMR (101 MHz, acetone D_6_) δ (ppm): 173.53 (-**C**OOCH_2_-), 97.55 (-O**C**(CH_3_)_2_O-), 65.39 (-O**C**H_2_-), 58.64 (-COO**C**H_2_-, d, *J* = 1.7 Hz), 51.67 (-PO(O**C**H_3_)_2_, d, *J* = 6.3 Hz), 41.49 (-**C**(CH_3_)COO-), 25.08 (-**C**H_2_PO(OCH_3_)_2_), 23.70 and 22.58 (-**C**H_3_ of acetonide), and 17.93 (-**C**H_3_). ^31^P NMR (162 MHz, acetone D_6_) δ (ppm): 28.87. FT-IR (cm^−1^): 2957(ν_(CH)_), 1733 (ν_(C=O)ester_), 1218 (ν_(P=O)_), and 1019 (ν_(P-O)_).

### 2.5. Synthesis of 2-(Dimethoxyphosphoryl)ethyl 3-hydroxy-2-(hydroxymethyl)-2-methylpropanoate (**3**)

The phosphonate compound **2** (16.0 g, 0.0516 mol), resin Dowex^®^50WX8 hydrogen form (50–100 mesh, 8.00 g), and methanol (180.0 mL) were added in a round bottom flask 250 mL equipped with a magnetic stirrer. After that, the suspended mixture was stirred at room temperature for 7 h under argon. The resin was then filtered out and washed with methanol. The filtered solution was concentrated under reduced pressure to yield the yellowish oil in quantitative yield. HRMS analysis (C_9_H_19_O_7_P): detected ion [M+H]^+^, calculated value *m*/*z*_calc_ = 271.0941 and experimental value *m*/*z*_exp_ = 271.0942.

^1^H NMR (400 MHz, CDCl_3_) δ (ppm): 4.42 (-COOC**H**_2_-, dt, *J* = 21.7, 6.2 Hz), 3.83 (HOC**H**_2_-, *J* = 11.5 Hz), 3.76 (-PO(OC**H**_3_)_2_, d, *J* = 10.9 Hz), 3.72 (HOC**H**_2_-, *J* = 11.5 Hz), 2.17 (-C**H**_2_PO(OCH_3_)_2_, dt, *J* = 17.9, 6.2 Hz), and 1.07 (-C**H**_3_, s). ^13^C NMR (101 MHz, CDCl_3_) δ (ppm): 175.39 (-**C**OOCH_2_-), 67.70 (-**C**H_2_OH), 58.17 (-COO**C**H_2_-, d, *J* = 7.3 Hz), 52.81 (-PO(O**C**H_3_)_2_, d, *J* = 6.7 Hz), 49.92 ((OHCH_2_)_2_**C**(CH_3_)COO-), 24.81 (-**C**H_2_PO(OCH_3_)_2_, d, *J* = 144.3 Hz), and 17.33 (-**C**H_3_). ^31^P NMR (162 MHz, CDCl_3_) δ (ppm): 30.36. FT-IR (cm^−1^): 3389(ν_(OH)_), 2957(ν_(CH)_), 1730 (ν_(C=O)ester_), 1218 (ν_(P=O)_), and 1019 (ν_(P-O)_).

### 2.6. Synthesis of 2-(Dimethoxyphosphoryl)ethyl 5-methyl-2-oxo-1,3-dioxane-5-carboxylate (**4**)

In a round bottom flask (250 mL) equipped with a magnetic stirrer, ethyl chloroformate (8.47 mL, 8.86 × 10^−2^ mol) was added dropwise to a stirred solution of **3** (12.00 g, 4.44 × 10^−2^ mol) in dry THF (80.0 mL) at 0 °C under argon atmosphere. After this addition was complete, a solution of triethylamine (TEA, 12.50 mL, 8.98 × 10^−2^ mol) in THF (20.0 mL) was slowly injected into the reaction flask. The solution was stirred under argon at 0 °C for 2 h and then stirred at room temperature for 16 h. The white solid was removed through filtration. The filtered solution was then concentrated under reduced pressure to obtain a viscous oil. The crude oil was purified using silica gel column chromatography with acetonitrile (100%) as the eluting solvent to yield a viscous oil (6.75 g). Yield: 51%. HRMS analysis (C_10_H_17_O_8_P): detected ion [M+H]^+^, calculated value *m*/*z*_calc_ = 297.0734 and experimental value *m*/*z*_exp_ = 297.0732.

^1^H NMR (400 MHz, CDCl_3_) δ (ppm): 4.71 (-OC(O)OC**H**_2_-, d, *J* = 10.9 Hz), 4.44 (-COOC**H**_2_, dt, *J* = 15.1, 7.1 Hz), 4.19 (-OC(O)OC**H**_2_-, d, *J* = 10.9 Hz), 3.76 (-PO(OC**H**_3_)_2_, dd, *J* = 10.9, 2.0 Hz), 2.18 (-C**H**_2_PO(OCH_3_)_2_, dt, *J* = 18.8, 7.1 Hz), and 1.34 (-CH_3_). ^13^C NMR (101 MHz, CDCl_3_) δ (ppm): 170.84 (-**C**OOCH_2_-), 147.36 (-O**C**(O)O-), 72.87 (-**C**H_2_OC(O)O**C**H_2_-), 60.05 (-COO**C**H_2_-, d, *J* = 2.7 Hz), 52.61(-PO(O**C**H_3_)_2_, d, *J* = 6.6 Hz), 40.21 (-**C**(CH_3_)COO-), 24.82 (-**C**H_2_PO(OCH_3_)_2_, d, *J* = 142.4 Hz), and 17.52 (-**C**H_3_). ^31^P NMR (162 MHz, CDCl_3_) δ (ppm): 28.61. FT-IR (cm^−1^): 2957(ν_(CH)_), 1755 (ν_(C=O)carbonate_), 1732 (ν_(C=O)ester_), 1238 (ν_(P=O)_), and 1019 (ν_(P-O)_).

### 2.7. Synthesis of Diethyl (3-Azidopropyl)phosphonate (Azido-phosphonate)

Diethyl (3-bromopropyl)phosphonate (5.00 g, 19.31 × 10^−3^ mol) and DMF (15.0 mL) were added to a round-bottom flask and stirred at room temperature under argon. Then, sodium azide (NaN_3_, 3.00 g, 46.15 × 10^−3^ mol) was slowly added into the flask containing the reaction mixture. The suspension was stirred for 1 h at room temperature and then for 42 h at 40 °C. Multiple filtrations were performed to remove the solid from the mixture. The solution was then concentrated under reduced pressure to obtain the yellow oil (3.46 g, 97% *w*/*w* in DMF).

^1^H NMR (400 MHz, CDCl_3_) δ (ppm): 4.02-4.18 (-PO(OC**H**_2_CH_3_)_2_), 3.37 (N_3_C**H**_2_-, t, *J* = 6.5 Hz), 1.72–1.92 (N_3_CH_2_C**H**_2_C**H**_2_-), and 1.32 (-PO(OCH_2_C**H**_3_)_2_, t, *J* =7.1 Hz). ^31^P NMR (162 MHz, CDCl_3_) δ (ppm): 30.75.

### 2.8. Ring-Opening Polymerization of **4**

Benzyl alcohol (BnOH, 24.3 mg, 2.25 × 10^−4^ mol), phosphonate monomer **4** (2.00 g, 6.76 × 10^−3^ mol), and thiourea (TU, 106 mg, 2.86 × 10^−4^ mol) were placed in a vial equipped with a magnetic stirrer, sealed with a rubber septum and flushed under argon. Under argon, the vial was filled with DCM (3.0 mL) and stirred to obtain a homogeneous solution. Then, a deoxygenated solution of DBU catalyst (41.0 mg, 2.70 × 10^−4^ mol) in DCM (1.0 mL) was injected quickly into the vial. The polymerization was launched and carried out at room temperature in the presence of argon. After 100 min, benzoic acid (45.0 mg) was added to the vial to stop the polymerization. The crude product was dissolved with acetone. This solution was dialyzed using a dialysis membrane (3500 Da) against acetone/water (1/3 in *v*/*v*). The polymer was recovered by lyophilization and dried at 45 °C to obtain a gummy solid with *M*_n,SEC_ = 2500 g·mol^−1^, *Ð* = 1.22, and *DP*_n,NMR_ = 23. ^1^H NMR (400 MHz, CDCl_3_) δ (ppm): 7.31–7.41 (C_6_**H**_5_CH_2_-), 5.13 (C_6_H_5_C**H**_2_-), 4.12–4.50 ((-C**H**_2_-)_backbone_ and -COOC**H**_2_-), 3.71–3.80 (-PO(OC**H**_3_)_2_), 2.09–2.24 (-C**H**_2_PO(OCH_3_)_2_), and 1.18–1.25 (-C**H**_3_). ^31^P NMR (162 MHz, CDCl_3_) δ (ppm): 29.15–29.96.

### 2.9. A Typical Copolymerization of **4** and MPC

Benzyl alcohol (17.1 mg, 1.58 × 10^−4^ mol), **4** (0.953 g, 3.22 × 10^−3^ mol), MPC monomer (0.621 g, 3.14 × 10^−3^ mol), and thiourea (TU, 71.8 mg, 1.94 × 10^−4^ mol) were placed in a vial equipped with a magnetic stirrer and sealed with a rubber septum and flushed under argon. Under argon, the vial was filled with DCM (3.0 mL) and stirred to obtain a homogeneous solution. Then, a deoxygenated solution of DBU catalyst (29.3 mg, 1.93 × 10^−4^ mol) in DCM (1.0 mL) was injected quickly into the vial. The polymerization was started and carried out at room temperature under argon. After 45 min, an excess of Dowex^®^50WX8 resin was added to the vial to quench the polymerization. The resin was filtered out using a syringe with filter 0.45 μm and concentrated under vacuum. The crude solid was washed with cold methanol and dried at 45 °C to yield the final polymer as a sticky solid with *M*_n,SEC_ = 3800 g·mol^−1^, *Ð* = 1.34.

^1^H NMR (400 MHz, CDCl_3_) δ (ppm): 7.28–7.41 (C_6_**H**_5_CH_2_-), 5.14 and 5.12 (C_6_H_5_C**H**_2_-), 4.67–4.78 (-C**H**_2_C≡CH), 4.15–4.53 ((-C**H**_2_-)_backbone_ and -COOC**H**_2_-), 3.71–3.81 (-PO(OC**H**_3_)_2_), 2.45–2.62 (-C≡C**H**), 2.06–2.30 (-C**H**_2_PO(OCH_3_)_2_), and 1.14–1.44 (-C**H**_3_). ^31^P NMR (162 MHz, CDCl_3_) δ (ppm): 28.62 and 29.14–30.06.

### 2.10. Synthesis of Alkyne-Functionalized Polycarbonate

The alkyne-functionalized polycarbonate (alkyne-PC) was synthesized using the same copolymerization protocol with [MPC]_0_:[BnOH]_0_:[TU]_0_:[DBU]_0_ = 45:1:1.21:1.19. The final polymer was characterized using NMR spectroscopy and SEC analysis with *M*_n,SEC_ = 4200 g·mol^−1^, *Ð* = 1.40, and *DP*_n, NMR_ = 46.

^1^H NMR (400 MHz, CDCl_3_) δ (ppm): 7.31–7.41 (C_6_**H**_5_CH_2_-), 5.15 (C_6_H_5_C**H**_2_-), 4.67–4.78 (-COOC**H**_2_-), 4.13-4.46 (-C**H**_2_-)_backbone_, 2.44–2.57 (-C≡C**H**), and 1.21–1.38 (-C**H**_3_).

### 2.11. Click Synthesis of Phosphonated-Functionalized Polycarbonate

Alkyne-polycarbonate polymer (poly(MPC)_46_, *M*_n,SEC_ = 4200 g·mol^−1^, *Ð* = 1.40, *M*_n,NMR_= 9220 g·mol^−1^, 0.43 g, 4.66 × 10^−5^ mol), diethyl (3-azidopropyl)phosphonate (97% *w*/*w* in DMF, 0.806 g, 3.54 × 10^−3^ mol), PMDETA (0.039 g, 2.25 × 10^−4^ mol), and DMF (3.0 mL) were added in a vial and sealed with a rubber septum. The solution was then deoxygenated for 30 min by bubbling argon at room temperature. Under flux argon, the deoxygenated solution was transferred to a second deoxygenated vial containing copper (I) bromide (CuBr, 29.2 mg, 2.03 × 10^−4^ mol) and a magnetic stirrer. The reaction was started and stirred at room temperature for 24 h under argon. The crude product was dissolved with acetone. This solution was dialyzed against acetone/water (2/3 in *v*/*v*) for 1 day and acetone for 3 days using a dialysis membrane (3500 Da). The polymer was recovered by removing the solvents under reduced pressure and drying it at 45 °C with *M*_n,SEC_ = 6000 g·mol^−1^ and *Ð* = 1.38.

^1^H NMR (400 MHz, acetone D_6_) δ (ppm): 8.08 (C**H**)_triazole_, 7.35–7.47 (C_6_**H**_5_CH_2_-), 5.27–5.32 (-COOC**H**_2_-), (5.18 (C_6_H_5_C**H**_2_-), 4.51–4.64 (-C**H**_2_CH_2_CH_2_PO(OEt)_2_), 4.26–4.39 (-C**H**_2_-)_backbone_, 3.98–4.14 (-PO(OC**H**_2_CH_3_)_2_), 2.14–2.26 and 1.70–1.85 (-C**H**_2_C**H**_2_PO(OEt)_2_), and 1.18–1.35 (-PO(OCH_2_C**H**_3_)_2_ and -C**H**_3_). ^31^P NMR (162 MHz, acetone D_6_) δ (ppm): 30.06.

## 3. Results and Discussion

### 3.1. Synthesis of Phosphonate-Functionalized Cyclic Carbonate Monomer

**Scheme 1:**
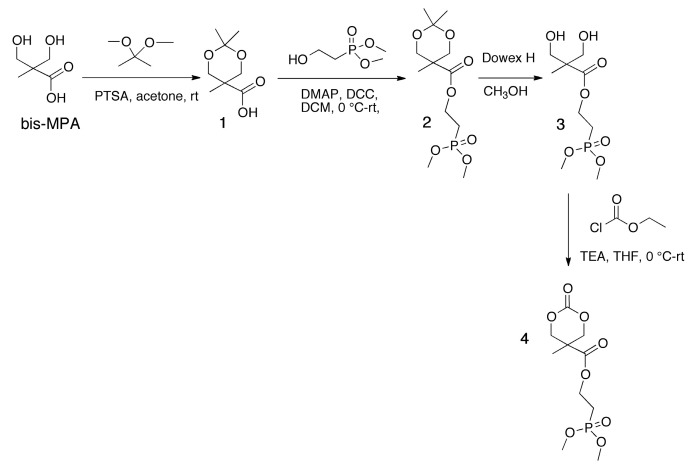
Synthetic route of the phosphonate-functionalized cyclic carbonate monomer.

To synthesize the phosphonate-functionalized polycarbonate, a novel cyclic carbonate monomer bearing a phosphonate group was synthesized from 2,2-bis(hydroxymethyl)propionic acid (bis-MPA) as the starting substance according to the synthetic route shown in Scheme 1.

**Figure 1:**
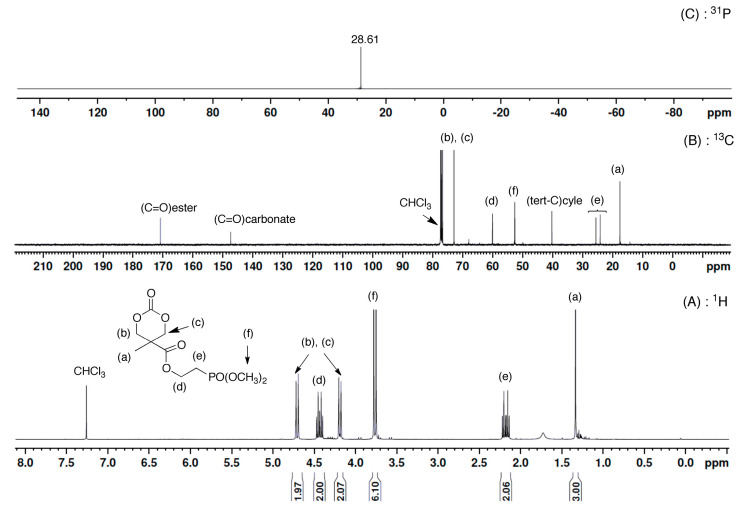
Overlaid ^1^H (**A**), ^13^C (**B**), and ^31^P (**C**) NMR spectra of **4** in CDCl_3_.

The synthesis of 2-(dimethoxyphosphoryl)ethyl 5-methyl-2-oxo-1,3-dioxane-5-carboxylate monomer (phosphonate-carbonate, **4**) was achieved in four steps with an average yield of 28.6%. After purification, the structure of the product was verified using NMR spectroscopy. Figure 1A depicts the ^1^H NMR spectrum of **4**, which exhibits characteristic signals at 4.19 ppm and 4.71 ppm (labeled (b), (c)), corresponding to the methylene of cyclic carbonate (–C**H**_2_OC(O)OC**H**_2_–) with the signal at 3.76 ppm, corresponding to the methoxy of phosphonate (–PO(OC**H**_3_)_2_). In addition, the presence of carbonate and phosphonate functionalities was also checked using ^13^C and ^31^P NMR spectroscopies with the presence of the carbonyl signal at 147.36 ppm as well as the phosphorous signal at 28.61 ppm, respectively (Figure 1B,C). These results demonstrate the effective synthesis of phosphonate-containing cyclic carbonate **4**.

Owing to the six-membered cyclic carbonate with high reactivity toward ring-opening polymerization, **4** could be an excellent monomer to synthesize well-defined phosphonate-functionalized polycarbonate. As a result, we attempted to polymerize **4** using the organocatalyzed ring-opening polymerization (ROP) technique to produce the corresponding phosphonate-functionalized polycarbonate.

### 3.2. ROP Synthesis of Well-Defined Phosphonate-Functionalized (Co)polycarbonates

**Scheme 2:**
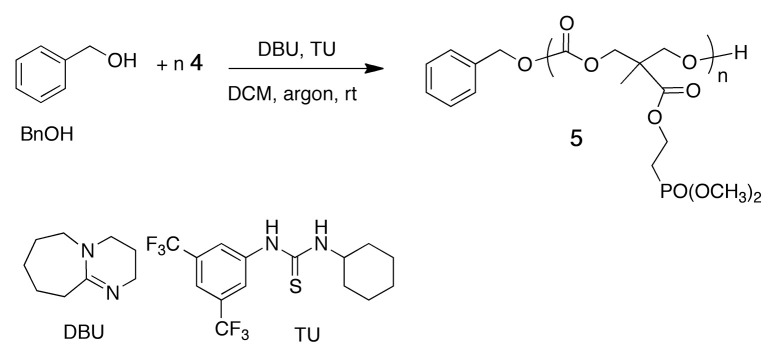
Organocatalyzed ROP of **4** with DBU/TU as the catalytic system in dichloromethane.

Due to its catalytic efficiency in the ROP of a wide range of cyclic monomers, DBU has been widely used, and is one of the most desired catalysts for ROP of functional cyclic carbonates [16,22]. In combination with the DBU catalyst, the addition of thiourea TU (co)catalyst serves as an activating monomer additive by increasing the electrophilicity of the carbonyl groups in cyclic carbonate monomers [23,24]. This favors the addition of a nucleophile to the monomer over the polymer backbone, accelerating the polymerization and minimizing the transesterification at high monomer conversion during the ROP [25]. Hence, the ROP of **4** was performed in the presence of the DBU/TU as catalytic systems and benzyl alcohol (BnOH) as the initiator in DCM to obtain the corresponding phosphonate functionalized-polycarbonate (Scheme 2) (Table 1, entry 1).

**Figure 2:**
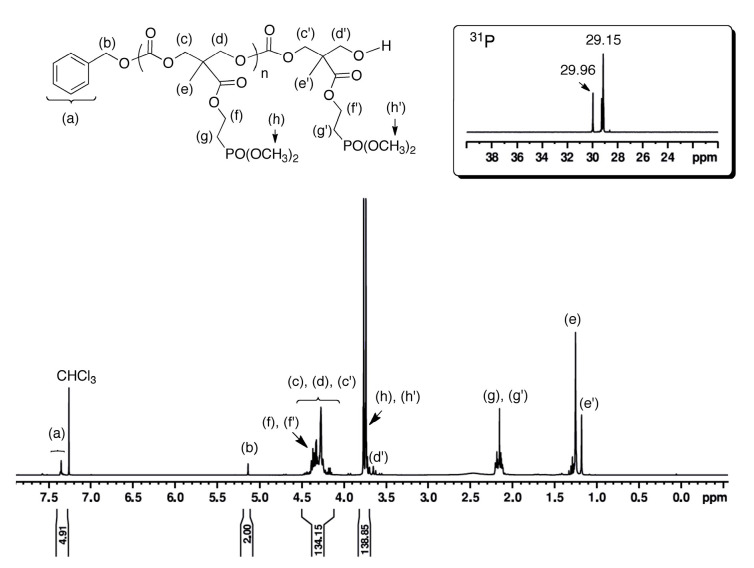
Overlaid ^1^H (**bottom**) and ^31^P NMR (**top right corner**) spectra of the phosphonate-functionalized polycarbonate **5** with *DP*_n,NMR_ = 23 and *Ð =* 1.22.

As expected, the polymerization of **4** was successful, with 90% of the monomer converted into polymer after 100 min. Monomer conversion was calculated based on the comparison of ^1^H NMR integral intensities between the methylene signal of cycle carbonate at 4.71 ppm and signal at 3.71-3.80 ppm due to the methoxy group (–PO(OC**H**_3_)_2_) of the monomer and the polymers formed. Furthermore, the formation and structure of phosphonate-functionalized polycarbonate **5** (poly[(**4**)], Scheme 2) were validated using ^1^H and ^31^P NMR spectroscopies. The ^1^H NMR spectrum of **5** (Figure 2) displays the characteristic polymer signals at 7.31–7.78 ppm and 5.13 ppm due to the benzyl extremity (C_6_**H**_5_C**H**_2_–, labeled (a) and (b)), followed by the polymer backbone signal at 4.12–4.50 ppm, and the methoxy (–PO(OC**H**_3_)_2_, labeled (h, h’)) at 3.71–3.80 ppm. The intensity integration ratio of these signals served to determine the degree of polymerization (*DP*_n,NMR_ = 23) and thus the average number of molecular weight by NMR (*M*_n,NMR_ = 6920 g·mol^−1^). The good correlation between the *M*_n,NMR_, and its theoretical value (*M*_n,th_ = 8100 g·mol^−1^) demonstrates the high polymer chain-end functionality and the control character of the polymerization. Additionally, the intact phosphonate group through the polymerization was validated by the phosphorous signals at 29.15–29.96 ppm on the ^31^P NMR spectrum (Figure 2).

**Figure 3:**
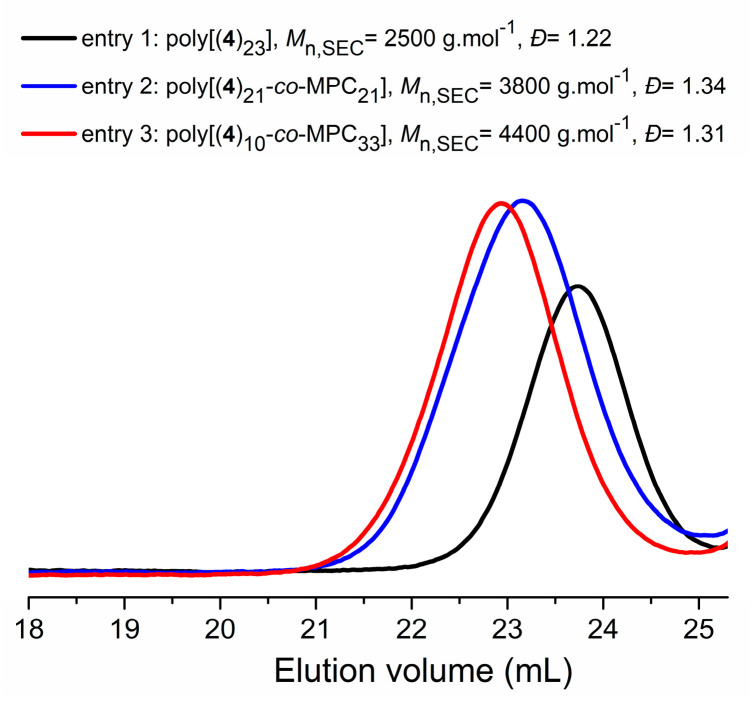
Overlaid SEC traces of the phosphonate-functionalized (co)polycarbonates synthesized in Table 1.

The presence of several phosphorous peaks indicates that the transesterification on phosphonate groups occurred during the polymerization. Indeed, the intramolecular and intermolecular transesterification reactions are frequently observed during the ROP of cyclic phosphoester monomers [26,27,28]. In our case, a possible transesterification through dimethyl phosphonate function could take place due to lower steric hindrance of the methoxy group (–OCH_3_). In addition, because of the high affinity of the TU catalyst with carbonyl (–C=O) and –P=O groups that are both present in **4,** this may result in a competitive interaction between these two functionalities and the TU. Such interaction could then decrease the performance of the TU catalyst. Despite the transesterification, the SEC analysis (Figure 3) reveals a unimodal and narrow dispersity corresponding to a polymer with *M*_n,SEC_ equal to 2500 g·mol^−1^. Moreover, the low dispersity value (*Ð* = 1.22) indicates the homogeneity of the resulting polymer chains. The difference value between the *M*_n,SEC_ and *M*_n,th_ could be due to the different hydrodynamic volumes of polymers and poly(methyl methacrylate) standards used for calibration. All these results demonstrate, for the first time, the successful synthesis of well-defined phosphonate-functionalized polycarbonate using organo-catalyzed ROP.

As a versatile method, the phosphonate-functionalized polycarbonate could directly be obtained using ROP of **4** or via ring-opening copolymerization of **4** with another comonomer. In particular, copolymerization has been employed to combine the properties of different materials to create novel materials. Copolymerization is therefore a simple method for expanding the series of biodegradable phosphonate-functionalized PC. In addition, we believe that the presence of comonomer units in the polymer backbone can significantly increase the steric hindrance surrounding the phosphonate groups, thereby limiting transesterification through the methoxy of phosphonates.

**Scheme 3:**
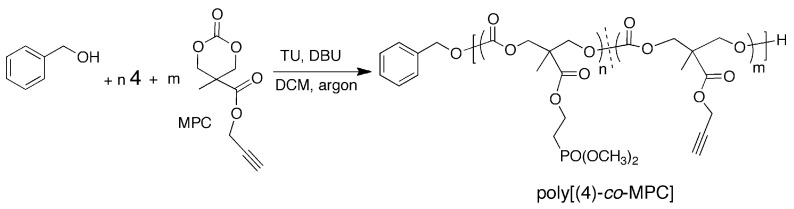
ROP of **4** and MPC monomer with DBU/TU as the catalytic system in DCM.

Then, the polymerization of **4** and alkyne-based cyclic carbonate (MPC) as a comonomer in different molar fractions was carried out with DBU/TU as the catalytic system at room temperature in DCM (Scheme 3). These polymerization conditions and the resulting polymer properties are summarized in Table 1 (entries 2 and 3).

**Figure 4:**
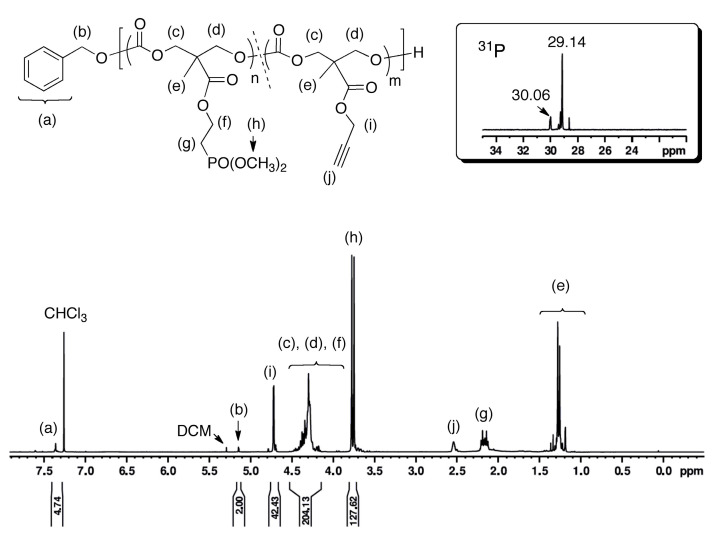
Overlaid ^1^H (**bottom**) and ^31^P NMR (**top-right corner**) spectra of phosphonate-functionalized poly[(**4**)_21_-*co*-MPC_21_] copolymer in CDCl_3_.

The successful copolymerization synthesis of phosphonate-functionalized (co)polycarbonates (poly[(**4**)-*co*-MPC], Scheme 3) was confirmed using SEC analysis with *M*_n,SEC_ equal to 3800 g·mol^−1^ and 4400 g·mol^−1^ and *Đ* values around 1.3 (Table 1, entries 2–3). The structure of the novel copolymers was confirmed with the appearance of typical signals such as the methoxy (labeled (h)) due to **4** units at 3.71–3.81 ppm along with the methylene (labeled (i)) signals at 4.67–4.78 ppm, attributed to MPC units in the ^1^H NMR spectrum (Figure 4). The composition of these resulting polymers was also identified as poly[(**4**)_21_-*co*-MPC_21_] and poly[(**4**)_10_-*co*-MPC_33_] using ^1^H NMR analysis of polymer chain-end. In addition, Table 1 shows the excellent correlation between the *M*_n,NMR_ and theoretical values (*M*_n,th_), indicating the controlled ROP process. Moreover, a low transesterification was confirmed by observing the intensity of phosphorous signals at 29.14 ppm and 30.06 ppm on the ^31^P NMR spectrum (top-right, Figure 4). These data demonstrate the successful copolymerization synthesis of well-defined phosphonate-functionalized (co)polycarbonates. Moreover, the convincing results indicate that the copolymerization of **4** with other monomers could be a relevant strategy for the development of novel phosphorous materials based on phosphonate-functionalized polycarbonates.

### 3.3. Post-Polymerization Synthesis of Phosphonate-Functionalized Polycarbonate

**Scheme 4:**
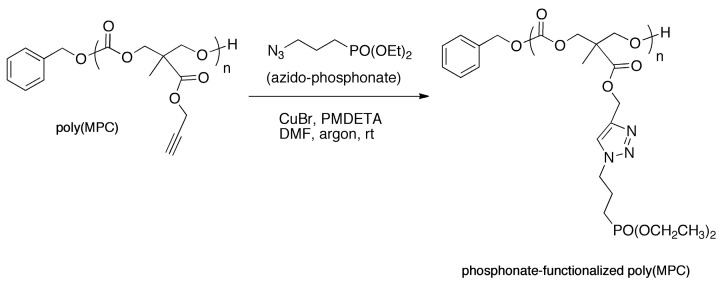
Post-polymerization synthesis of the phosphonate-functionalized polycarbonate.

Although the synthesis of well-defined phosphonate-functionalized polycarbonates has been successfully demonstrated using (co)polymerizations of **4**, the multi-step monomer synthesis and the transesterification of the phosphonate group during the polymerization are the major drawbacks when it comes to considering a scale-up. These limitations motivated us to develop a robust and highly efficient post-polymerization method to prepare the phosphonate-functionalized PC. Unlike the low yield of the previously mentioned “amine-acid” reaction, the “click” reaction has emerged as the most efficient and powerful reaction for the synthesis of functional polymers [29,30,31]. “Click” chemistry was chosen to synthesize the phosphonate-functionalized PC using the reaction between an alkyne-functionalized polycarbonate based on poly(MPC)_46_ (*M*_n,SEC_ = 4200 g·mol^−1^, *Ð* = 1.4) and an azido-phosphonate compound in the presence of CuBr and PMDETA as the catalyst system and DMF (Scheme 4).

**Figure 5:**
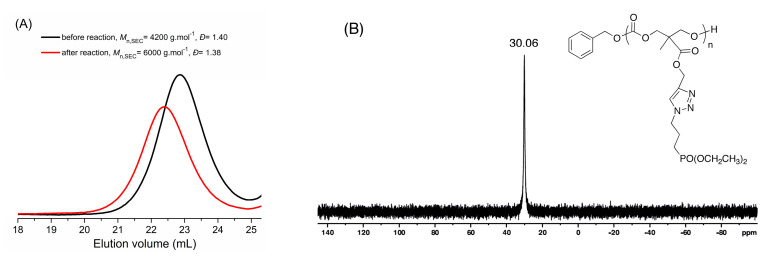
(**A**) Overlaid SEC traces of poly(MPC)_46_ before and after the “click” reaction; (**B**) ^31^P NMR spectrum of poly(MPC)_46_ after the “click” reaction in acetone D_6_.

The SEC trace of the resulting product shifted to the lower retention volume, indicating the higher molecular weight of the polymer product and also the successful grafting of azido-phosphonate on the polymer backbone (Figure 5A). In addition, the existence of a single phosphorous signal at 30.06 ppm on the ^31^P NMR spectrum confirmed the effective incorporation of phosphonate into poly(MPC)_46_ chains (Figure 5B).

**Figure 6:**
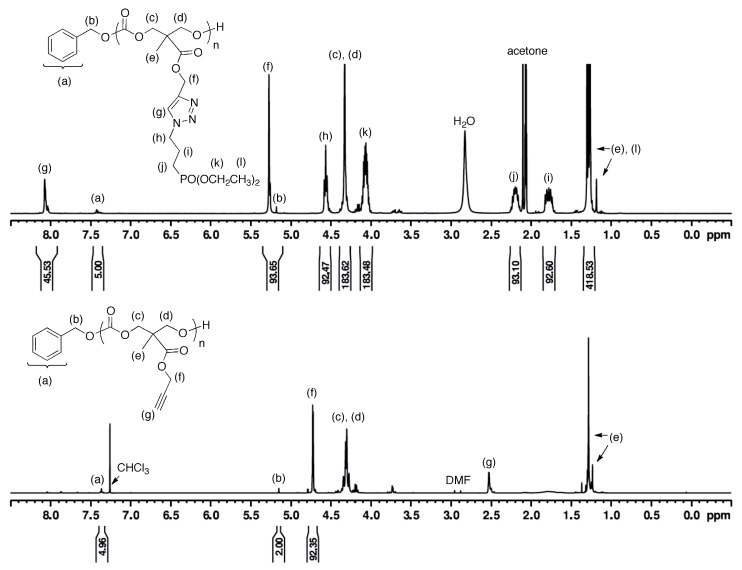
Overlaid ^1^H NMR spectra of poly(MPC)_46_ in CDCl_3_ before and after the “click” reaction with azido-phosphonate in acetone D_6_.

Moreover, the overlaid ^1^H NMR spectra of poly(MPC)_46_ before and after the reaction (Figure 6) upheld the formation of the phosphonate-functionalized poly(MPC)_46_ product with the appearance of the novel signal at 8.08 ppm (labeled (g)), characteristic of the proton of cyclic triazole along with the methylene signals (labeled (h), (i) and (j)) due to grafted azido-phosphonate on the polymer backbone. The functionality yield was also determined to be above 95%. All these results confirm the high efficiency of the “click” reaction to graft the azido-phosphonate on the polycarbonate backbone as well as the successful synthesis of phosphonate-functionalized polycarbonate.

## 4. Conclusions

We have successfully demonstrated two strategies for synthesizing polycarbonate-bearing pendant phosphonate groups. For the first time, a well-defined phosphonate-functionalized polycarbonate was directly synthesized using organocatalyzed ROP of new phosphonate containing cyclic carbonate. The copolymerization of phosphorus monomer was also performed to access the various structures of polycarbonate-containing phosphonate groups. Furthermore, copolymerization could be a useful approach for limiting the transesterification of the dimethyl phosphonate group. Finally, “click” chemistry was effectively used to graft the phosphonate group on the polycarbonate backbone. This post-modification approach could be a convenient way to avoid the undesirable transesterification of the phosphonate group during the ROP process.

## Figures and Tables

**Table 1:** Summary of ROP of **4** and its copolymerization with MPC monomer using TU/DBU.

Entry	[M]_0_:[BnOH]_0_:[TU]_0_:[DBU]_0_	4/MPC ^a^	Time (min)	Conv. ^b^ (%)	*M*_n,th_^c^ (g·mol^−1^)	*M*_n,NMR_^d^ (g·mol^−1^)	*M*_n,SEC_^e^ (g·mol^−1^)	*Ð* ^e^
1	30:1:1.27:1.20	100/0	100	90	8100	6920	2500	1.22
2	40:1:1.23:1.22	50/50	45	100	9990	10480	3800	1.34
3	41:1:1.23:1.22	22/78	45	100	9110	9600	4400	1.31

^a^ Molar percentage of **4** and MPC. ^b^ Determined by ^1^H NMR spectroscopy of crude polymer solution. ^c^ *M*_n,th_ = (Conv./100) × [(*DP*_n,th of **4**_ × 296) + (*DP*_n,th of MPC_ × 198)] + 108. ^d^ Determined by ^1^H NMR spectroscopy of polymer. ^e^Determined by size exclusion chromatography with DMF containing NaNO_3_ (50 mM) as an eluent and PMMA standards used for the calibration curve.

## Data Availability

Data are available upon requests made to the corresponding authors.
